# Activation of the heat shock transcription factor Hsf1 is essential for the full virulence of the fungal pathogen *Candida albicans*

**DOI:** 10.1016/j.fgb.2010.08.010

**Published:** 2011-03

**Authors:** Susan Nicholls, Donna M. MacCallum, Florian A.R. Kaffarnik, Laura Selway, Scott C. Peck, Alistair J.P. Brown

**Affiliations:** a School of Medical Sciences, University of Aberdeen, Institute of Medical Sciences, Foresterhill, Aberdeen AB25 2ZD, United Kingdom; b The Sainsbury Laboratory, Norwich Research Park, Colney Lane, Norwich NR4 7UH, United Kingdom; c Division of Biochemistry, 271H Bond Life Sciences Center, University of Missouri-Columbia, Columbia, MO 65211, USA

**Keywords:** *Candida albicans*, Heat shock, Hsf1, Transcriptional activation, Phosphorylation, Pathogenesis

## Abstract

The evolutionarily conserved heat shock transcription factor Hsf1 plays a central role in thermal adaptation in the major fungal pathogen of humans, *Candida albicans*. Hsf1 becomes hyperphosphorylated in response to heat shock and activates the transcription of genes with heat shock elements (HSEs) in their promoters, these genes contributing to thermal adaptation. However, the relevance of Hsf1 activation to *C. albicans* virulence is not clear as this pathogen is thought to be obligately associated with warm blooded animals, and this issue has not been tested because *HSF1* is essential for viability in *C. albicans*. In this study, we demonstrate that the HSE regulon is active in *C. albicans* cells infecting the kidney. We also show the CE2 region of Hsf1 is required for activation and that the phosphorylation of specific residues in this domain contributes to Hsf1 activation. *C. albicans HSF1* mutants that lack this CE2 region are viable. However, they are unable to activate HSE-containing genes in response to heat shock, and they are thermosensitive. Using this *HSF1* CE2 deletion mutant we demonstrate that Hsf1 activation, and hence thermal adaptation, contributes significantly to the virulence of *C. albicans*.

## 1 Introduction

The major fungal pathogen of humans, *Candida albicans,* exists in many healthy individuals as a relatively harmless commensal organism in the microflora of the oral cavity and the gastrointestinal and urogenital tracts (Odds, 1988; Calderone, 2002). However, *C. albicans* is also a frequent cause of mucosal infections such as oral thrush and vaginitis (Ruhnke, 2002). These infections generally arise when host immune defenses are weakened or the local microflora is disturbed. In patients with severely compromised immune defenses, e.g. in chemotherapy or transplant patients, *C. albicans* can establish potentially fatal systemic infections of the bloodstream and of major organs such as the kidney, liver and brain (Filler and Kullberg, 2002; Kullberg and Filler, 2002).

A combination of virulence factors and fitness attributes contribute to the pathogenicity of *C. albicans.* Virulence attributes are thought to impact directly upon interactions between the fungus and its host (Odds et al., 2003). For example, yeast-hypha morphogenesis balances the context-dependent requirements of the fungus for rapid growth, nutrient foraging, dissemination and invasion (Gow et al., 2002, 2003; Sundstrom, 2006). The secretion of aspartyl proteinases and (phospho)lipases enhance invasion and nutrient provision (Hube and Naglik, 2002). Also, a battery of adhesins promotes attachment to host tissues (Staab et al., 1999; Hoyer et al., 2008), whilst invasins enhance uptake by host cells (Phan et al., 2007).

In contrast, fitness attributes enhance the physiological robustness of *C. albicans* cells in the diverse niches they can occupy within the host. For example, effective metabolic adaptation enhances nutrient assimilation and growth (Brown, 2005), whereas robust stress responses are thought to help protect the fungus against environmental insults and host immune defences (Quinn and Brown, 2007). Oxidative, nitrosative, osmotic and thermal stress responses in particular have attracted significant interest in the field (San Jose et al., 1996; Alonso-Monge et al., 1999; Smith et al., 2004; Ullmann et al., 2004; Hromatka et al., 2005; Chauhan et al., 2006; Enjalbert et al., 2006; Brown et al., 2009).

The generally held view is that fungal virulence factors and fitness attributes have evolved relatively rapidly, allowing species to adapt to the diverse niches they occupy. With regard to stress regulation in the fungi, sensors and upstream signalling components display a high degree of evolutionary divergence, as do the transcription factors that drive the corresponding adaptive responses (Butler et al., 2009; Nikolaou et al., 2009; Rispail et al., 2009). Also, while core stress regulatory modules are highly conserved, in some cases their cellular roles have diverged. For example, while AP1-like transcription factors such as Yap1, Cap1 and Pap1 drive transcriptional responses to oxidative stress (Cohen et al., 2002; Chen et al., 2008; Znaidi et al., 2009), the stress activated protein kinases Hog1 and Sty1 mediate responses to different types of stress in evolutionarily divergent yeasts (Chen et al., 2003; O’Rourke and Herskowitz, 2004; Enjalbert et al., 2006). Nevertheless, the downstream adaptive responses driven by these pathways are well conserved. For example, glycerol is generally accumulated as an osmolyte during adaptation to osmotic stress, and thioredoxin and glutaredoxin systems are induced to mediate redox adaptation in response to reactive oxygen species.

Historically, the heat shock response in *C. albicans* has been of interest because, although additional factors probably influence morphogenesis in the host, temperature up-shifts are associated with the yeast-to-hyphal transition (Swoboda et al., 1995; Shapiro et al., 2009) and because Hsp90 fragments are immuno-protective in systemic candidiasis (Matthews et al., 1991). We became interested in the heat shock response and its regulation in *C. albicans* because this pathogen is thought to be obligately associated with warm blooded animals (Odds, 1988). We confirmed that *C. albicans* does activate a *bona fide* transcriptional response to an acute heat shock (Enjalbert et al., 2003; Nicholls et al., 2009), and that an evolutionarily conserved heat shock transcription factor, Hsf1, plays a central role in this response (Nicholls et al., 2009). We also demonstrated that Hsf1 becomes hyperphosphorylated in response to heat shock, that Hsf1 activates transcription via heat shock elements (HSEs) in the promoters of its target genes, and that transcriptional activation by Hsf1 is specific for heat shock and is not triggered by other medically relevant stresses (Nicholls et al., 2009).

Why then has a heat shock response been retained in this pathogen during evolution if it is obligately associated with warm blooded animals? We reasoned that the primary role of Hsf1 in *C. albicans* *in vivo* might be to mediate thermal homeostasis rather than adaptation to acute heat shocks. In other words, Hsf1 might play an essential role *in vivo* by tuning the levels of essential *C. albicans* chaperones such as Hsp70, Hsp90 and Hsp104 to the growth temperature of the host niche. This is not possible to test using *hsf1/hsf1* null mutants because *HSF1* is essential for viability in *C. albicans* (Nicholls et al., 2009). Therefore, in this study we have tested this hypothesis by generating *C. albicans HSF1* mutants in which basal Hsf1 activity is retained, but heat shock activation has been blocked. We show that a specific domain of *C. albicans* Hsf1 is required for heat shock activation and that phosphorylation at specific sites within this domain contribute to Hsf1 activation. We confirm that HSE-containing genes are activated in *C. albicans* during systemic infection of the kidney and that Hsf1 activation is required for the full virulence of *C. albicans.* Hence, thermal homeostasis is integral to the adaptation of this fungus during pathogenesis.

## 2 Materials and methods

### 2.1 Strains and growth conditions

*C. albicans* strains used in this study are listed in Table 1. Strains were grown in yeast–peptone–dextrose (YPD) medium (Sherman, 1991). The expression of tetracycline-regulated *HSF1* alleles was down-regulated by the addition of doxycycline to a final concentration of 20 μg/ml for at least 6 h (Nicholls et al., 2009). To heat stress *C. albicans,* cells were grown in YPD at 30 °C for at least 6 h to mid-exponential phase, and then rapidly transferred to pre-warmed flasks at 45 °C for 30 min.

### 2.2 Strain construction

Doxycycline-conditional *C. albicans* *HSF1* mutants were generated as previously described for CLM62-1 (Table 1) (Nicholls et al., 2009). *HSF1* truncation mutants were created using the mini Ura-blaster cassette (Wilson et al., 2000) in an *hsf1/HSF1* background (CLM61-1: Nicholls et al., 2009). Briefly, *HSF1::hisG-URA3-hisG* truncation cassettes were created by PCR amplification using the primers HSF1-CTMt-F, HSF1-CTMt-R, HSF1-CE2t-F and HSF1-CE2t-R (Table 2). These cassettes were then transformed into *C. albicans* CLM61-1 to delete codons 566–762 of the *HSF1* open reading frame to create the allele CE2t (strain SN127), and to delete codons 732–762 of *HSF1* to generate the allele CTMt (strain SN128: Table 1). *Ura3-*minus segregant of SN127 and SN128 were then selected by growth on media containing 5-fluoroorotic acid (Wilson et al., 2000), and the genotypes of these strains confirmed by PCR diagnosis. The HSE-lacZ and Basal-lacZ reporters (Nicholls et al., 2009) were then transformed into these *C. albicans* strains selecting for the *URA3* marker (Murad et al., 2000). Correct integration was confirmed by PCR diagnosis with oligonucleotides RPS1-GEN and LacZ-F (Table 2).

### 2.3 Plasmid construction

Hsf1 phosphorylation mutants were created by mutagenesis of plasmid pACT1pHSF1 (Nicholls et al., 2009). First, the CE2 domain was deleted (codons 564–578) by inverse PCR using oligos CE2 del F/R (Table 2) thereby introducing an internal BamHI site. The resultant plasmid was called pΔCE2. Second, oligonucleotides All WT-F/R (Table 2) were annealed and cloned into this new BamHI site to recreate the wild type sequence (pSDM-WT). Third, oligonucleotides All E-F/R and All A-F/R (Table 2) were annealed and cloned into the BamHI site to generate *HSF1* alleles with either S571E, T575E, S577E, T578E mutations (pSDM-E) or S571A, T575A, S577A, T578A mutations (pSDM-A). These plasmids were integrated into *C. albicans* CAI4 selecting for the *URA3* marker, and into *C. albicans* CLM62-1 using the *NAT1* marker. Correct genomic integration was confirmed by PCR using oligos D1, D2, HSF-F and RPS1-GEN (Table 2).

### 2.4 mRNA analyses

Published methods were used for RNA preparation, and northern analyses of the *ACT1*, *HSP90* and *HSP104* mRNAs (Wicksteed et al., 1994; Brown et al., 2001). A non-radioactive kit was used for detection of the probe (ECL Direct™ Nucleic Acid Labelling and Detection Systems, Amersham, UK) (Nicholls et al., 2009).

qRT-PCR was used to quantify the levels of the *lacZ, HSP90*, *HSP104*, *ENO1* transcripts relative to the internal *ACT1* mRNA control using the primers listed in Table 2. RNA samples (2 μg) were incubated in 20 μl reactions with 1.5 μl of DNase I, 1.5 μl of RNase OUT, 2 μl of DNase I buffer (Invitrogen; Paisley, UK) at room temperature for 15 min. cDNA was then prepared using Superscript II (Invitrogen) as per the manufacturer’s protocols. Real-time (RT)-PCR SYBR green (Roche; Welwyn Garden City, UK) assays were carried out as per the manufacturer’s instructions on a Roche LightCycler® 480.

### 2.5 Reporter assays

LacZ expression levels were assayed in quadruplicate on independent transformants as described previously (Rupp, 2002; Nicholls et al., 2009). Briefly, *C. albicans* cells were grown for at least 6 h to exponential phase. Half of each culture was subjected to a stress for 30 min and the other half acted as the untreated control. Cells were harvested and resuspended in 1 ml of Z buffer (60 mM Na_2_HPO_4_, 40 mM NaH_2_PO_4_ 10 mM KCl, 1 mM MgSO_4_, 50 mM β-mercaptoethanol), and then 50 μl of chloroform and 20 μl of 1% SDS were added. Samples were equilibrated at 37°C for 10 min, and then reactions started by addition of 200 μl of pre-warmed OPNG (4 mg/ml). Samples were incubated until a yellow colour developed whereupon the reaction was stopped by addition of 0.4 ml of 1 M Na_2_CO_3_. β-galactosidase activities were measured in Miller Units.

### 2.6 Protein extraction and western blots

Protein extracts were prepared and subjected to Western blotting using published protocols (Smith et al., 2004). Briefly, cells were resuspended in 250 μl lysis buffer (0.1 M Tris–HCl, pH 8, 10% glycerol, 1 mM DTT, pepstatin A, Protease Inhibitor Cocktail) and sheared with glass beads in a Mini-bead beater (6 × 30 s with 1 min intervals on ice). Lysates were centrifuged at 13,000 rpm for 10 min at 4 °C. Protein extracts were dephosphorylated using lambda phosphorylase (NEB) as per the manufacturer’s protocols. Protein extracts (15 g) were subjected to SDS–PAGE electrophoresis, blotted for 2 h at 30 V, and membranes blocked for at least 1 h at room temperature using 5% milk. Membranes were probed overnight at 4 °C with a rabbit anti-FLAG-HRP conjugated antibody (diluted 1/200,000) (Sigma). Membranes were then washed and signals detected with an HRP Western blotting kit (Amersham, UK).

### 2.7 Virulence assays and *in vivo* samples for qRT-PCR

All animal experimentation conformed to the requirements of United Kingdom Home Office legislation and of the Ethical Review Committee of the University of Aberdeen.

The virulence of *C. albicans* strains SC5314, CE2t, CTMt and Basal-lacZ (Table 1) was assessed in the mouse intravenous challenge model of systemic candidiasis (MacCallum et al., 2010). Female BALB/c mice (6–8 weeks old; Harlan, UK) were infected intravenously with a saline suspension of *C. albicans* cells (5.6–5.8 × 10^4^ CFU/g mouse body weight) from cultures grown overnight in NGY medium (MacCallum and Odds, 2005). After 72 h of infection, animals were weighed, humanely terminated, and their kidneys removed aseptically. One kidney from each animal was used to assay fungal burdens. Infection Outcome Scores were calculated on the basis of fungal burdens and animal weights, as described previously (MacCallum et al., 2010). The second kidney from each animal was snap frozen for RNA extraction and qRT-PCR analysis (Walker et al., 2009).

To analyse gene expression *in vivo*, *C. albicans* strains SC5314, Basal-lacZ and HSE-lacZ (Table 1) were used to initiate infections in mice. For each strain, a saline suspension (2.4–4.0 × 10^4^ CFU/g mouse body weight), prepared from overnight cultures in NGY medium, was used to intravenously infect six female BALB/c mice (6–8 weeks old; Harlan, UK). Mice were monitored daily and culled when they showed signs of illness and/or when they had lost 20% body weight. Mice were humanely terminated and the kidneys aseptically removed. One kidney was used for fungal burden determination and the other was snap frozen in liquid nitrogen for subsequent RNA extraction and qRT-PCR analyses (Walker et al., 2009).

For virulence assays, kidney fungal burdens and infection outcome scores were compared by Kruskal–Wallis and Mann–Whitney U tests. All statistic analyses were carried out using SPSS Statistics package 17.0.

## 3 Results

### 3.1 The CE2 domain is required for Hsf1-mediated transcriptional activation

**Fig. 1:**
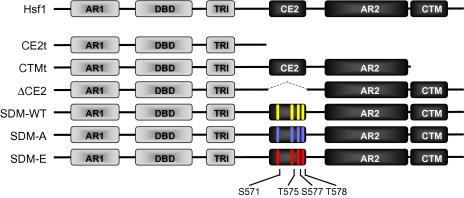
Construction of *C. albicans HSF1* mutants. The top line illustrates the domain structure of *C. albicans* Hsf1, roughly to scale, by analogy to *S. cerevisiae* Hsf1. Hsf1 domains include two transcriptional activation regions (AR1 and AR2), a DNA-binding domain (DBD), a trimerisation region (TRI), a control element (CE2) and a carboxy-terminal region (CTM). Schematic representations of *C. albicans* mutations created in this study lie below the wild type Hsf1 structure (Hsf1). Truncations of the CTM region (CTMt) and all of the carboxy-terminus up to and including the CE2 region (CE2t) were created at the *HSF1* locus in an *HSF1/hsf1* heterozygote, these constructs being expressed from the endogenous *HSF1* promoter. Hsf1 phosphorylation sites in the CE2 region are highlighted in yellow. *Bam*HI sites were introduced on each site of the CE2 region to create the wild type control (SDM-WT) for the subsequent site directed mutagenesis of these sites to alanine, in blue (SDM-A) or glutamate, in red (SDM-E). The CE2 region was also deleted from SDM-WT to create the mutation ΔCE2. These constructs were expressed from the *ACT1* promoter (Section 2).

Eukaryotic heat shock transcription factors are well conserved and have similar structures. *Saccharomyces cerevisiae* Hsf1 is an 833 amino acid protein that contains a winged-helix-turn-helix DNA-binding domain (DBD: residues 168–284), a hydrophobic repeat region necessary for coiled-coil formation during Hsf1p trimerization (residues 326–424), amino-terminal and carboxy-proximal transcriptional activation domains (AR1 and AR2: residues 1–424 and 583–782, respectively), a negative regulatory domain (CE2: residues 535–550), and a carboxy-terminal modulator domain that alleviates the repression by CE2 in response to heat shock (CTM: residues 783–833) (Santoro et al., 1998; Sakurai and Fukasawa, 2001; Hashikawa and Sakurai, 2004). *C. albicans* Hsf1 displays homology to these domains (Fig. 1).

Our first aim was to test the functional significance of the putative regulatory domains located in the carboxy-terminal half of *C. albicans* Hsf1. Therefore we set out to create *HSF1* truncation mutants in which the CTM domain alone, the AR2 and CTM domains together, and then all three carboxy-terminal domains (CE2-AR2-CTM) were selectively deleted. The approach was to create the truncations by integrating a *URA3* cassette at the 3′ end of the remaining wild type allele in the *hsf1/HSF1* heterozygote, CLM61-1 (Table 1). Strains carrying the CTM (CTMt) and CE2-AR2-CTM (CE2t) truncations were successfully generated (Fig. 1), but parallel attempts to generate an AR2-CTM truncation were not.

**Fig. 2:**
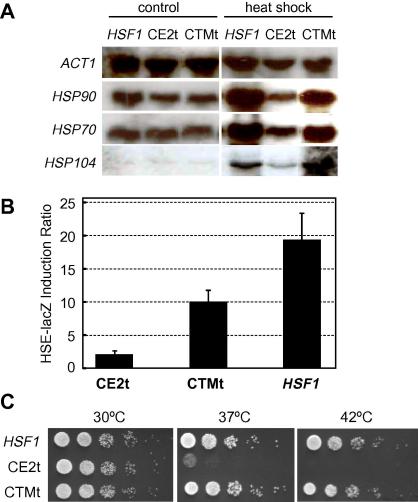
Removal of the CE2 and AR2 regions attenuates the ability of Hsf1 to activate the HSE regulon in response to heat shock and confers thermosensitivity. (A) Northern analysis of the *HSP70, HSP90* and *HSP140* mRNAs using *ACT1* as an internal loading control. RNA was isolated from *C. albicans* strains *hsf1/HSF1* (CLM61-1), CE2t (SN127) and CTMt (SN128) (Table 1) grown at 30 °C in YPD to exponential phase and then subjected to a 30 to 45 °C heat shock for 30 min, or maintained at 30 °C (control). (B) Activation of the HSE-lacZ reporter was measured by assaying β-galactosidase activities in heat shocked *C. albicans hsf1/HSF1,* CE2t and CTMt cells expressing either the basal-lacZ or HSE-lacZ reporter (SN65, SN66, SN138, SN141, SN148, and SN151; Table 1). Fold-induction in HSE-lacZ expressing cells is shown relative to equivalent cells expressing the basal-lacZ reporter. (C) Impact of Hsf1 truncations upon thermotolerance. *C. albicans* cells were serially diluted, spotted onto YPD, and incubated overnight at 30 °C, 37 °C or 42 °C: *HSF1, hsf1/HSF1* (CLM61-1); CE2t (SN127); CTMt (SN128).

We then tested the impact of the CTMt and CE2t truncations upon Hsf1 function. Hsf1 is known to mediate the activation of HSE-containing genes in response to heat shock (Nicholls et al., 2009). Therefore, we compared *HSP70*, *HSP90* and *HSP104* mRNA levels in strains expressing wild type and truncated forms of Hsf1 by northern analysis (Fig. 2A). As expected, untreated cells expressing wild type Hsf1 expressed *HSP70* and *HSP90* at high basal levels, and the levels of these mRNAs increased following heat shock (30 °C to 45 °C for 30 min). *HSP104* mRNA levels were below detectable levels in untreated cells and were significantly induced in heat shocked cells. These data are entirely consistent with our previous observations (Nicholls et al., 2009). Interestingly, truncation of the CTM domain did not affect the basal levels of the *HSP70*, *HSP90* or *HSP104* mRNAs, nor did it impair the ability of Hsf1 to increase their expression in response to heat shock (Fig. 2A). This suggests that the CTM domain is not required to alleviate repression of Hsf1 activity in *C. albicans*, in contrast to the situation in *S. cerevisiae* (Sakurai and Fukasawa, 2001). In contrast the CE2t truncation attenuated the heat shock induction of the *HSP70*, *HSP90* and *HSP104* mRNAs.

This finding was assessed further by assaying the activation of the HSE-lacZ reporter in *HSF1,* CE2t and CTMt cells (Fig. 2B). The heat shock activation of this HSE-lacZ reporter in *C. albicans* has been shown to be dependent upon Hsf1 (Nicholls et al., 2009). Not surprisingly, therefore, this reporter was strongly activated in response to heat shock in cells expressing wild type Hsf1. Some attenuation of this activation was observed following truncation of the CTM domain. However, activation was almost completely inhibited in cells expressing the CE2 truncation, thereby confirming that Hsf1 is unable to activate HSE-containing promoters following deletion of the CE2, AR2 and CTM domains.

Taken together our data suggested that sequences in the CE2-AR2 region are required for Hsf1-mediated transcriptional activation in response to heat shock. To test whether deletion of the CE2-AR2 region also alters the thermotolerance of *C. albicans*, strains expressing wild type or truncated versions of Hsf1 were grown at different temperatures (Fig. 2C). Cells expressing wild type or CTMt versions of Hsf1 were able to grow up to 42 °C (the maximum temperature tested at which wild type cells grew). In contrast cells expressing the CE2 truncation were thermo-sensitive. Therefore, there was a strong correlation between the ability of Hsf1 molecules to activate transcription in *C. albicans* and the thermotolerance of the corresponding cells. This result is consistent with the behaviour of Hsf1 truncations in *S. cerevisiae*, where removal of sequences after Hsf1 residue 583 blocks Hsf1 activation and renders cells temperature sensitive for growth at 37 °C (Morano et al., 1999).

### 3.2 Phosphorylation within the Hsf1 CE2 domain affects thermotolerance

*C. albicans* Hsf1 is known to be activated by hyperphosphorylation in response to heat shock (Nicholls et al., 2009), but the residues phosphorylated in response to heat shock have not yet been identified. Recently, two independent studies of the *C. albicans* phosphoproteome have identified four residues within the CE2 domain of Hsf1 that can be phosphorylated: S571, T575, S577 and T578 (Beltrao et al., 2009; Kaffarnik and Peck, unpublished). The impact of heat shock on Hsf1 phosphorylation was not investigated in these studies. Nevertheless, phosphorylation at these residues might contribute to the heat shock activation of Hsf1.

**Fig. 3:**
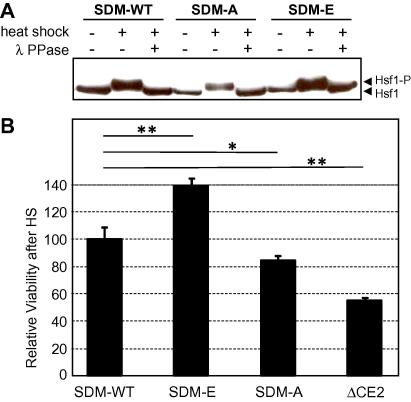
Influence of Hsf1 phosphorylation sites upon Hsf1 phosphorylation and thermotolerance in *C. albicans*. (A) Mutagenesis of putative phosphorylation sites at S571, T575, S577 and S578 does not prevent Hsf1 phosphorylation. *C. albicans* cells expressing different FLAG-tagged Hsf1 mutants were subjected to a 30 to 45 °C heat shock or maintained at 30 °C, and protein extracts analysed by Western blotting with an anti-FLAG antibody: SDM-WT, SN250; SDM-A, SN251; SDM-E, SN252 (Table 1). Some extracts were also treated with λ phosphatase to confirm that the observed band shifts represented Hsf1 phosphorylation. Bands corresponding to phosphorylated (Hsf1-P) and unphosphorylated Hsf1 are highlighted by arrows on the right. (B) Mutations in the Hsf1 CE2 domain affect thermotolerance. *C. albicans hsf1/tet-HSF1* cells (CLM62-1) transformed with various plasmids were grown for 6 h in the presence of 20 μg/ml doxycycline to down-regulate Hsf1, subjected to a 30 to 45 °C heat shock for 30 min and then viability assayed by plating on YPD and determining colony forming units (CFU): SDM-WT (SN254); SDM-A (SN255); SDM-E (SN256); ΔCE2 (SN257) (Table 1). Viability was measured relative to heat stressed control cells (SDM-WT, SN254). Means and standard deviations from triplicate experiments are shown: ^*^p < 0.05; ^**^p < 0.01.

To test this possibility, the corresponding *HSF1* codons were all mutated either to alanine (SDM-A) or glutamate codons (SDM-E) to generate non-phosphorylatable and phosphomimetic versions of Hsf1, respectively. Cells expressing these alleles were compared with cells expressing an equivalent wild type construct (SDM-WT: Fig. 1). These constructs were expressed with amino-terminal FLAG tags to permit detection by immunoblot analysis (Section 2). Immunoblotting revealed that, as expected the FLAG-tagged wild type Hsf1 was phosphorylated in response to heat shock (Fig. 3A). The SDM-A and SDM-E also showed gel shifts following heat shock that were resolved by treatment with λ phosphatase. Therefore, further residues in *C. albicans* Hsf1, in addition to S571, T575, S577 and T578, are phosphorylated in response to heat shock.

We tested whether mutagenesis of the Hsf1 phosphosites at S571, T575, S577 and T578 affects the thermotolerance of *C. albicans* by examining cell viability after a 30–45 °C heat shock (Fig. 3B). The SDM-WT, SDM-A and SDM-E *HSF1* alleles were transformed into the doxycycline-conditional *hsf1/tet-HSF1* mutant (CLM62-1) to generate strains SN254-257 (Table 1). Cells were grown in YPD containing doxycycline at 30 °C for 6 h to down-regulate the *tet-HSF1* conditional allele (Nicholls et al., 2009). Cells were then either subjected to heat shock from 30 °C to 45 °C for 30 min, or maintained at 30 °C as a control, and then cell viabilities measured by plating (Fig. 3B). Cells expressing the phosphomimetic SDM-E allele displayed a significant increase in heat resistance compared to the SDM-WT control, whereas cells expressing the non-phosphorylatable SDM-A allele showed a small but statistically significant decrease in thermotolerance. These results suggest that, although additional Hsf1 residues appear to be phosphorylated in response to heat shock, the observed phosphorylation at S571, T575, S577 and T578 contributes to thermotolerance in *C. albicans.*

### 3.3 Deletion of the Hsf1 CE2 domain blocks Hsf1 phosphorylation and confers thermosensitivity upon *C. albicans*

**Fig. 4:**
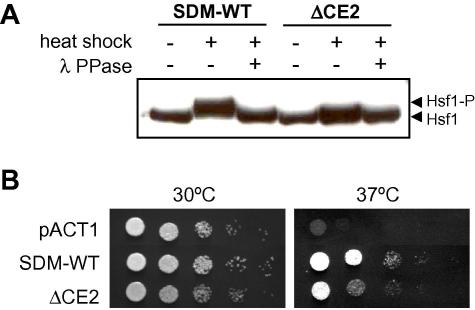
Deletion of the CE2 domain blocks detectable Hsf1 phosphorylation and renders *C. albicans* thermosensitive. (A) CE2 deletion blocks Hsf1 phosphorylation. *C. albicans* cells expressing FLAG-tagged versions of Hsf1 were heat shocked or maintained at 30 °C, and protein extracts examined by western blotting: SDM-WT*,* SN250; ΔCE2, SN253 (Table 1). (B) The CE2 domain is required for thermotolerance. Serially diluted *C. albicans hsf1/tet-HSF1* cells (CLM62-1) expressing different *HSF1* alleles, or the empty pACT1 vector as a control, were spotted onto YPD containing doxycycline and incubated overnight at 30 °C or 37 °C.

To test whether the additional phosphorylation sites in Hsf1 are located within the CE2 domain, we generated a new *HSF1* allele that lacks this domain (ΔCE2: Fig. 1). This *HSF1* allele was expressed with an analogous amino-terminal FLAG tag to the SDM alleles described above (Section 2). Immunoblot analysis of the wild type SDM-WT control once again confirmed that Hsf1 is phosphorylated in response to heat shock (Fig. 4A). In contrast, no detectable phosphorylation of Hsf1 lacking the CE2 domain, was observed, suggesting that most, if not all, of the phosphorylated residues lie within this domain.

We tested whether deletion of the Hsf1 CE2 domain has a concomitant effect upon thermotolerance. This was the case when growth of the ΔCE2 mutant was compared with the wild type control on plates at 37 °C (Fig. 4B) and when their viabilities were compared after a 30–42 °C heat shock (Fig. 3B). Significantly, the viability of the ΔCE2 mutant (in which Hsf1 phosphorylation was blocked) was lower than the SDM-A mutant (in which some Hsf1 phosphorylation was retained) (Fig. 3B). Therefore, all of the mutants we examined exhibited a strong correlation between the extent of Hsf1 phosphorylation and the degree of thermotolerance. These results reinforce the idea that thermal adaptation in *C. albicans* is dependent upon Hsf1 activation by phosphorylation in the CE2 domain.

### 3.4 The C. albicans Hsf1-HSE regulon is activated during systemic infection of the kidney

Is the Hsf1-HSE regulon required to drive thermal homeostasis during *C. albicans* infections? Our first step towards addressing this major question was to test whether the Hsf1-HSE regulon is expressed during systemic kidney infections by qRT-PCR.

**Fig. 5:**
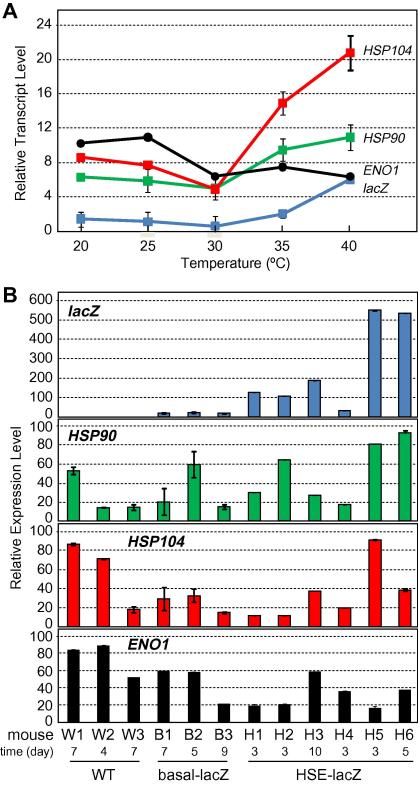
Expression of HSE-containing genes in *C. albicans* cells from infected mouse kidneys. *HSP90, HSP104, lacZ* and *ENO1* mRNA levels were measured relative to the internal *ACT1* mRNA control by qRT-PCR. (A) Control experiments assaying transcript levels in *C. albicans* SN1 cells (expressing HSE-lacZ) grown *in vitro* in YPD at different temperatures. Error bars are from triplicate assays from two independent qRT-PCR analyses on each sample. Similar results were obtained for two independent experiments. (B) *C. albicans* mRNA levels in cells from infected kidneys of mice with systemic candidiasis: W1–W3, three different mice infected with SC5314; B1–B3, three mice infected with SN1 (expressing basal-lacZ); H1–H6, six mice infected with SN2 (expressing HSE-lacZ). The time post-infection (in days) that each mouse was sacrificed is indicated.

Our starting point involved the calibration of our qRT-PCR quantification methods using *C. albicans* cells grown at defined temperatures *in vitro*. *C. albicans* cells expressing HSE-lacZ in a wildtype *HSF1/HSF1* background (SN2: Table 1) were grown at 20 °C, 25 °C, 30 °C, 35 °C or 40 °C. RNA was isolated from these cultures and the expression of the *lacZ,* *HSP90* and *HSP104* mRNAs measured relative to the internal *ACT1* mRNA control. In addition, we examined *ENO1* mRNA levels (relative to *ACT1*) as a non-heat shock inducible control. *lacZ* mRNA levels increased when the growth temperature increased above 30 °C (Fig. 5A), which was entirely consistent with the growth temperature-related increase in β-galactosidase expression levels that we observed previously (Nicholls et al., 2009). The qRT-PCR data confirmed that the expression of this Hsf1-dependent reporter is modulated at the transcript level in response to growth temperature. Furthermore, analogous increases in the levels of the Hsf1-regulated transcripts *HSP90* and *HSP104* were observed at growth temperatures above 30 °C (Fig. 5A). Indeed the correlation between the *lacZ,* *HSP90* and *HSP104* transcript levels was very strong (Table 3). In contrast, *ENO1* mRNA levels did not increase with growth temperature (Fig. 5A), and the correlation between *ENO1* and *HSP104* transcript levels, for example, was weak (−0.43 *in vitro,* and −0.13 *in vivo*). Data from independent experiments confirmed the reproducibility of these observations, thereby reinforcing the idea that the Hsf1-HSE regulon tunes the expression of essential chaperones to the growth temperature of *C. albicans*.

Next we examined *lacZ,* *HSP90*, *HSP104* and *ENO1* transcript levels *in vivo,* in *C. albicans* cells from infected kidneys. Mice were sacrificed between days 3–10 after being injected intravenously with *C. albicans* SC5314 (*HSF1/HSF1* control), SN1 (*HSF1/HSF1* cells containing Basal-lacZ) or SN2 (*HSF1/HSF1* cells containing HSE-lacZ) (Table 1), once infection had progressed to a point when high kidney fungal burdens were obtained (kidney fungal burdens = 6.1 ± 0.7 log_10_ CFU/g). We used the clinical isolate, SC5314, as the infection control in this experiment because the virulence of this strain has been shown to be equivalent to *C. albicans* CAI4 cells carrying CIp10 (Walker et al., 2009), the vector used to generate pBasal-lacZ and pHSE-lacZ (Nicholls et al., 2009). To maintain the *in vivo* transcriptome, kidneys were rapidly dissected, snap frozen and the tissues fixed before RNA preparation (Walker et al., 2009: Materials and methods). Transcript levels were then measured by qRT-PCR relative to the internal *ACT1* mRNA control (Fig. 5B).

As expected, no detectable *lacZ* mRNA was seen in *C. albicans* SC5134 cells, and minimal *lacZ* mRNA levels were observed in SN1 cells (Basal-lacZ) from kidney infections in independent animals (Fig. 5B). In contrast significant *lacZ* mRNA levels were observed in SN2 cells (HSE-lacZ) infecting the kidney, indicating that the Hsf1-HSE regulon is activated in *C. albicans* during systemic infections. Also *HSP90* and *HSP104* were expressed during experimental infections with *C. albicans* SC5134 and SN1 (Fig. 5B). These *C. albicans* strains do not contain the HSE-lacZ reporter, but they do contain an intact Hsf1-HSE regulon. Therefore, these data strengthen the idea that the Hsf1-HSE regulon is activated during systemic infections of the kidney.

HSE-lacZ expression levels did vary significantly between individual infected animals. However, HSE-lacZ mRNA levels correlated well with the *HSP90* and *HSP104* transcripts in these infections (Fig. 5B; Table 3). In contrast the levels of these transcripts did not correlate with *ENO1* mRNA levels, indicating that this experimental variation was not due to technical noise but to *bona fide* regulation of the Hsf1-HSE regulon *in vivo*. This Hsf1-HSE regulon appears to be induced specifically in response to temperatures above about 30 °C (Nicholls et al., 2009). Taken together our data suggest variation in the temperatures of individual animals with systemic candidiasis. Such variation has been observed previously in animals with severe *C. albicans* infection (Spellberg et al., 2005).

### 3.5 Hsf1 activation contributes significantly to the virulence of C. albicans

The above observations suggested that thermal homeostasis might contribute significantly to the physiological fitness of *C. albicans* cells *in vivo*, and hence that Hsf1 might contribute to the virulence of this pathogen. However, *HSF1* is an essential gene in *C. albicans* (Nicholls et al., 2009) and therefore it was not possible to test this hypothesis using *hsf1/hsf1* null mutants. Nevertheless, having constructed a *C. albicans* mutant in which Hsf1 is not activated (CE2t) it became possible to test whether activation of the Hsf1-HSE regulon is required for virulence in *C. albicans*.

**Fig. 6:**
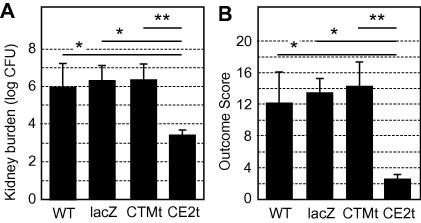
*C. albicans* mutants that are unable to activate Hsf1 display attenuated virulence in the mouse model of systemic candidiasis*.* (A) Kidney burdens measured at day 3: WT, SC5314; HSE-lacZ (SN2); CTMt (SN128); CE2t (SN127) (Table 1). (B) Infection Outcome Scores calculated after three days (means and standard deviation for data from six animals): ^∗^*p* < 0.05; ^∗∗^*p* < 0.01. Higher outcome scores reflect more severe infection.

Mice were infected with *C. albicans* SN127 (CE2t) and with the control strains SC5314 and SN128 (Table 1). The clinical isolate SC5314 represented a wild type control. SN128 expressed the heat shock-inducible Hsf1 CTMt truncation mutant, thereby controlling for the molecular manipulations used to generate the Hsf1 CE2t mutant. The challenge dose for the virulence assays was chosen to produce infection outcome scores of approximately 14 at day 3 post-infection for the wild type control strain (challenge dose = 5.6–5.8 × 104 CFU/g body weight). Virulence was assayed by determining infection outcome scores, based upon weight change and kidney burdens at 3 days post-infection (Materials and methods; MacCallum et al., 2010) (Fig. 6). The CTMt cells displayed similar fungal burdens and outcome scores to the wild type control, indicating that our molecular approaches *per se* had not impaired the virulence of *C. albicans.* Furthermore, inclusion of strain SN2 in these virulence experiments confirmed that the HSE-lacZ construct used in the above experiments (Section 3.4) did not affect the virulence of *C. albicans* (Fig. 6).

The CE2t mutant displayed significantly reduced fungal burdens and outcome scores compared with the control strains (Fig. 6). Furthermore, a 10-fold increase in challenge inoculum did not increase infection outcome score (not shown), demonstrating the severe virulence attenuation of this strain. This indicated that Hsf1 induction is required for the full virulence of *C. albicans.*

## 4 Discussion

In this study, we report two important conclusions relating to heat shock regulation in the fungal pathogen *C. albicans*. First, we conclude that the CE2 domain of the essential heat shock transcription factor, Hsf1, is required for its activation in response to heat shock. This CE2 domain lies between the trimerisation region and the activation domain AR2 of Hsf1 (Fig. 1). Mutations that remove this CE2 domain block heat shock activation of the *HSP70, HSP90* and *HSP104* mRNAs and of the Hsf1-dependent HSE-lacZ reporter (Fig. 2A and B), confer thermosensitivity upon *C. albicans* (Figs. 2C, 3B and 4B), and block the heat shock inducible hyperphosphorylation of Hsf1 (Fig. 4A). These data highlight the importance of the CE2 domain in the regulation of Hsf1 activity, and also demonstrate the importance of Hsf1 activation for thermal adaptation in *C. albicans.*

The CE2 domain harbours four amino acid residues at which phosphorylation has been detected in *C. albicans* cells: S571, T575, S577 and T578 (Beltrao et al., 2009; Kaffarnik and Peck, unpublished). However, these high throughput studies did not look at the proteome under heat shock conditions. Therefore, we tested whether phosphorylation at these residues contributes Hsf1 activation. We showed that blocking phosphorylation at S571, T575, S577 and T578 (SDM-A) attenuated the thermal resistance of *C. albicans* slightly, whereas phosphomimetic mutations at these sites (SDM-E) slightly increased thermotolerance (Fig. 3B). However, some Hsf1 phosphorylation was retained in these mutants (Fig. 3A), and deletion of the whole CE2 domain conferred a greater effect upon *C. albicans* thermotolerance than mutagenesis of S571, T575, S577 and T578 alone (Fig. 3B). We conclude that while phosphorylation at these four residues contributes to Hsf1 activation, additional phosphorylation sites exist within Hsf1, probably in the CE2 domain (e.g. at S567 and/or S573). However, we do not exclude the possibility that mutagenesis of CE2 inhibits phosphorylation elsewhere in Hsf1.

Our second important conclusion is that activation of the Hsf1-HSE regulon contributes significantly to the virulence of *C. albicans*. Two key observations underpin this conclusion. Firstly, preventing the activation of Hsf1 severely attenuates the virulence of *C. albicans* (Fig. 6). Deleting the Hsf1 CTM domain did not attenuate virulence (CTMt), whereas further truncation of CE2 and AR2 domains (CE2t) significantly attenuated virulence, although the slow growth of this latter mutant at 37 °C probably contributed to this phenotype (Fig. 2). Secondly, the Hsf1-HSE regulon is activated during systemic kidney infections (Fig. 5). Interestingly, there was variation in the degree of activation of the Hsf1-HSE regulon between experimental infections. Some of this variability may be intrinsic to the mice and in particular to the differing rates of progression in individual animals. This affected the times at which samples were taken for analysis post-infection, which might have contributed to this variation (Fig. 5). Nevertheless, this observation is consistent with variation in the temperatures of individual animals with systemic candidiasis, some animals being febrile, others not (Spellberg et al., 2005). Taken together, these data are entirely consistent with the hypothesis that Hsf1 is essential for thermal homeostasis in *C. albicans,* tuning the levels of essential chaperones to the temperature of the local microenvironment. This proposed function does not preclude a role for Hsf1 in protection against acute heat shocks in *C. albicans.* However, it is not clear when this pathogen might be exposed to the sudden and dramatic temperature up-shifts associated with the experimental heat shocks induced *in vitro,* given that *C. albicans* is thought to be obligately associated with warm blooded animals (Odds, 1988).

## Figures and Tables

**Table 1:** *C. albicans* strains.

Strain	Genotype	Source
SC5314	Clinical isolate	Gillum et al. (1984)
CAI4	*ura3::*λ *imm434/ura3::*λ *imm434*	Fonzi and Irwin (1993)
CLM60-1	*ade2::hisG/ade2::hisG, ura3::*λ *imm434/ura3::*λ *imm434, ENO1/eno1::ENO1-tetR-ScHAP4AD-3XHA-ADE2, hsf1*::*hisG-URA3-hisG/HSF1*	Nicholls et al. (2009)
CLM61-1	*ade2::hisG/ade2::hisG, ura3::*λ *imm434/ura3::*λ *imm434, ENO1/eno1::ENO1-tetR-ScHAP4AD-3XHA-ADE2, hsf1*::*hisG/HSF1*	Nicholls et al. (2009)
CLM62-1	*ade2::hisG/ade2::hisG, ura3::*λ *imm434/ura3::*λ *imm434, ENO1/eno1::ENO1-tetR-ScHAP4AD-3XHA-ADE2, hsf1::hisG/URA3-tetp-HSF1*	Nicholls et al. (2009)
SN1	*ura3::*λ *imm434/ura3::*λ *imm434, pBasal-lacZ(URA3)*	Nicholls et al. (2009)
SN2	*ura3::*λ *imm434/ura3::*λ *imm434, pHSE-lacZ(URA3)*	Nicholls et al. (2009)
SN65	*ade2::hisG/ade2::hisG, ura3::*λ *imm434/ura3::*λ *imm434, ENO1/eno1::ENO1-tetR-ScHAP4AD-3XHA-ADE2, hsf1*::*hisG/HSF1 pBasal-lacZ(URA3)*	Nicholls et al. (2009)
SN66	*ade2::hisG/ade2::hisG, ura3::*λ *imm434/ura3::*λ *imm434, ENO1/eno1::ENO1-tetR-ScHAP4AD-3XHA-ADE2, hsf1*::*hisG/HSF1, pHSE-lacZ(URA3)*	Nicholls et al. (2009)
SN127	*ade2::hisG/ade2::hisG, ura3::*λ *imm434/ura3::*λ *imm434, ENO1/eno1::ENO1-tetR-ScHAP4AD-3XHA-ADE2, hsf1*::*hisG/HSF1-CE2t-hisG-URA3-hisG*	This study
SN128	*ade2::hisG/ade2::hisG, ura3::*λ *imm434/ura3::*λ *imm434, ENO1/eno1::ENO1-tetR-ScHAP4AD-3XHA-ADE2, hsf1*::*hisG/HSF1-CTMt-hisG-URA3-hisG*	This study
SN138	*ade2::hisG/ade2::hisG, ura3::*λ *imm434/ura3::*λ *imm434, ENO1/eno1::ENO1-tetR-ScHAP4AD-3XHA-ADE2, hsf1*::*hisG/HSF1-CE2t-hisG pBasal-lacZ(URA3)*	This study
SN141	*ade2::hisG/ade2::hisG, ura3::*λ *imm434/ura3::*λ *imm434, ENO1/eno1::ENO1-tetR-ScHAP4AD-3XHA-ADE2, hsf1*::*hisG/HSF1-CE2t-hisG pHSE-lacZ(URA3)*	This study
SN148	*ade2::hisG/ade2::hisG, ura3::*λ *imm434/ura3::*λ *imm434, ENO1/eno1::ENO1-tetR-ScHAP4AD-3XHA-ADE2, hsf1*::*hisG/HSF1-CTMt-hisG pBASAL-lacZ(URA3)*	This study
SN151	*ade2::hisG/ade2::hisG, ura3::*λ *imm434/ura3::*λ *imm434, ENO1/eno1::ENO1-tetR-ScHAP4AD-3XHA-ADE2, hsf1*::*hisG/HSF1-CTMt-hisG pHSE-lacZ(URA3)*	This study
SN250	*ura3::*λ *imm434/ura3::*λ *imm434, pSDM-WT(URA3)*	This study
SN251	*ura3::*λ *imm434/ura3::*λ *imm434, pSDM-A(URA3)*	This study
SN252	*ura3::*λ *imm434/ura3::*λ *imm434, pSDM-E(URA3)*	This study
SN253	*ura3::*λ *imm434/ura3::*λ *imm434, pΔCE2(URA3)*	This study
SN254	*ade2::hisG/ade2::hisG, ura3::*λ *imm434/ura3::*λ *imm434, ENO1/eno1::ENO1-tetR-ScHAP4AD-3XHA-ADE2, hsf1::hisG/URA3-tetp-HSF1, pSDM-WT(NAT1)*	This study
SN255	*ade2::hisG/ade2::hisG, ura3::*λ *imm434/ura3::*λ *imm434, ENO1/eno1::ENO1-tetR-ScHAP4AD-3XHA-ADE2, hsf1::hisG/URA3-tetp-HSF1, pSDM-A(NAT1)*	This study
SN256	*ade2::hisG/ade2::hisG, ura3::*λ *imm434/ura3::*λ *imm434, ENO1/eno1::ENO1-tetR-ScHAP4AD-3XHA-ADE2, hsf1::hisG/URA3-tetp-HSF1, pSDM-E(NAT1)*	This study
SN257	*ade2::hisG/ade2::hisG, ura3::*λ *imm434/ura3::*λ *imm434, ENO1/eno1::ENO1-tetR-ScHAP4AD-3XHA-ADE2, hsf1::hisG/URA3-tetp-HSF1, pSDM-ΔCE2 (NAT1)*	This study

**Table 2:** Oligonucleotides used in this study.

Primer	Sequence (5′–3′)	Application
HSF-F	GTTTGTGGCACTGACAGA	Diagnosis of *HSF1* allele
HSF-Diag	GACTGTTATTAGCTGGGC	Diagnosis of *HSF1* allele
HSF1-CE2t	TCAGACTGCACAACCTACTTATGAATCACCATTATCAACCAGCGATACCAATAATAATAACAACAACAGTACCTTTGAATATCAACAAGCTGTCAATtaaGTTTTCCCAGTCACGACGTT	Disruption cassette for creating truncation
HSF1-CTMt	GCTACTACAACTCCTGGTTCTAATGTGCCAAATGGTGGCCATTATAATAATGGGAATATAAATTTTGTTGATTCACCAATTGCAATGACTCCAGGCTaAGTTTTCCCAGTCACGACGTT	Disruption cassette for creating truncation
HSF-C-Term-R	CGAGGTGAAAGAAAATGCTAGGCATTAGGTAGACTACAACAGATTGTATTTCGTAAACTTATTGTATTAAACTTAAAATTTATCTATATACCTAAACAACGTGTGGAATTGTGAGCGGATA	Disruption cassette for creating truncation
D1	GCACGGATTTGCTGATTTCAG	Diagnosis of Hsf1 truncation
D2	GTGAAATAGTCCGAACTACCC	Diagnosis of Hsf1 truncation
WT-F	GATCCGCACATTCAAGACGTCCAAGTATGTCTAGAACCAAATCTACAG	oligo to recreate CE2 domain
WT-R	GATCCTGTAGATTTGGTTCTAGACATACTTGGACGTCTTGAATGTGCG	oligo to recreate CE2 domain
ALL A-F	GATCCGCACATTCAAGACGTCCAGCTATGTCTAGAGCTAAAGCTGCTG	oligo to recreate CE2 domain with phosphosites changed to A
ALL A-R	GATCCAGCAGCTTTAGCTCTAGACATAGCTGGACGTCTTGAATGTGCG	oligo to recreate CE2 domain with phosphosites changed to A
ALL E-F	GATCCGCACATTCAAGACGTCCAGAAATGTCTAGAGAAAAAGAAGAAG	oligo to recreate CE2 domain with phosphosites changed to E
ALL E-R	GATCCTTCTTCTTTTTCTCTAGACATTTCTGGACGTCTTGAATGTGCG	oligo to recreate CE2 domain with phosphosites changed to E
CE2 DEL F	TATGGATCCCCAGAAGGGTCCATAGAAGAT	Inverse PCR oligo to delete CE2 domain
CE2 DEL R	TATGGATCCTCGGTTAGTCAACATGAGACG	Inverse PCR oligo to delete CE2 domain
RPS1-GEN	GTGTGGGATTAAGTGAATACG	Diagnosis of insertion of CIp10-based plasmids at RPS1
LACZ-F	GCTTCAAGGTTTTGGTTCTCC	Diagnosis of insertion of CIp10-based plasmids at RPS1
ACT1-F	GATGAAGCCCAATCCAAAAG	PCR-amplification of ACT1 probe
ACT1-R	GGAGTTGAAAGTGGTTTGGT	PCR-amplification of ACT1 probe
HSP90-F	TAGTCGACTATGGCTGACGCAAAAGTTG	PCR-amplification of *HSP90* probe
HSP90-R	ACATGGTACCACGACCCAAT	PCR-amplification of *HSP90* probe
HSP104-F	TTGCTGCATTTATCCCATCA	PCR-amplification of *HSP104* probe
HSP104-R	CAGCATCACCAATCAACACC	PCR-amplification of *HSP104* probe
HSP70-F	TGATGCTGCCAAGAATCAAG	PCR-amplification of *HSP70* probe
HSP70-R	TCACCAGCAGTGGCTTTAACT	PCR-amplification of *HSP70* probe
qENO1-F	AAAACCCAGAATCCGACCC	qRT-PCR oligo
qENO1-R	AAGCATCCCAGTCATCTTCAG	qRT-PCR oligo
qACT1-F	GCTGAACGTATGCAAAAG	qRT-PCR oligo
qACT1-R	GAACAATGGATGGACCAG	qRT-PCR oligo
qHSP90-F	CTGGTGCTGACGTTTCTA	qRT-PCR oligo
qHSP90-R	ACCAGCGTTAGATTCCCA	qRT-PCR oligo
qLACZ-F	CACCTCAAGTTCCTCAAGAA	qRT-PCR oligo
qLACZ-R	CCTACGAAGTTACCATTGAC	qRT-PCR oligo
qHSP104-F	GAAGGCTCAACACAGTATTT	qRT-PCR oligo
qHSP104-R	GGTCGTATTTCATCTGGAGG	qRT-PCR oligo

**Table 3:** Correlation coefficients for comparisons of *lacZ* mRNA levels with other transcripts.

Transcript	Expression *in vitro*	Expression *in vivo*
lacZ	1	1
HSP90	0.88	0.85
HSP104	0.93	0.80
ENO1	−0.46	−0.13

Correlation coefficients were calculated by comparing the relative expression level of *HSP* or *ENO1* transcripts with the corresponding level of the *lacZ* transcript. Correlation coefficients were calculated using *in vitro* data for each experimental condition examined (Fig. 5A), and *in vivo* data from each animal infected with *C. albicans* cells expressing HSE-lacZ (H1–H6 in Fig. 5B).
